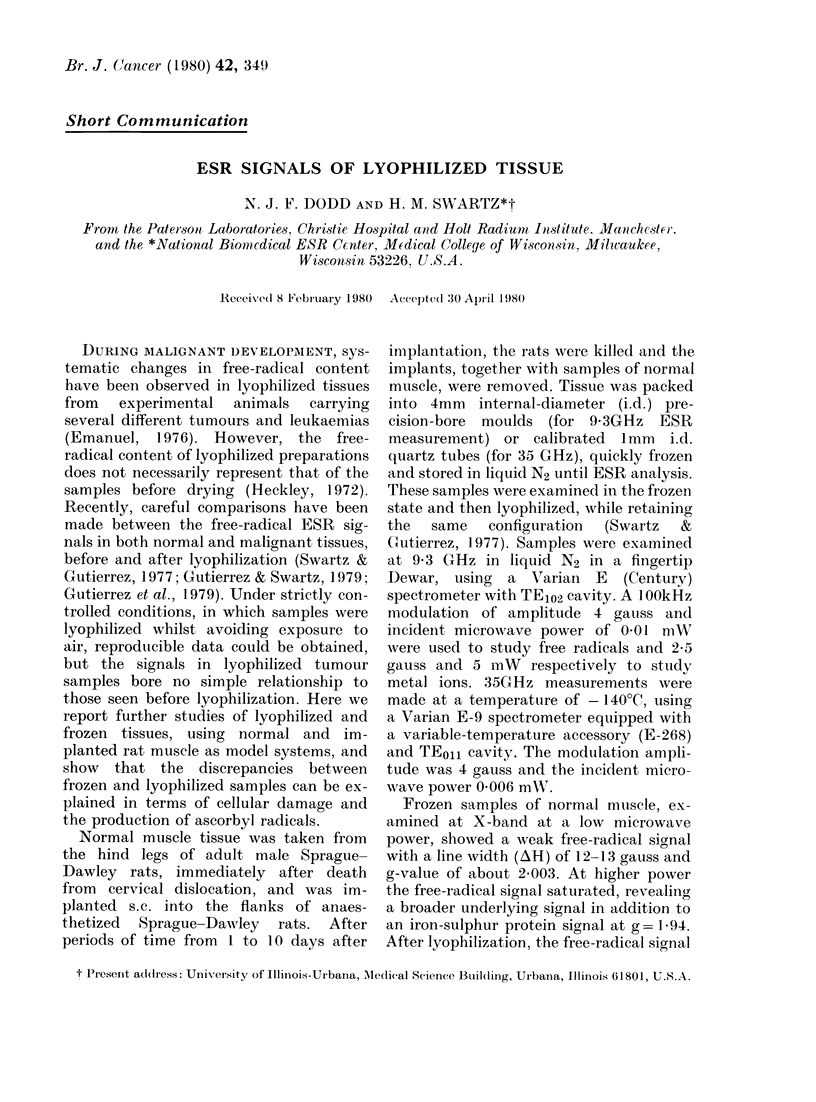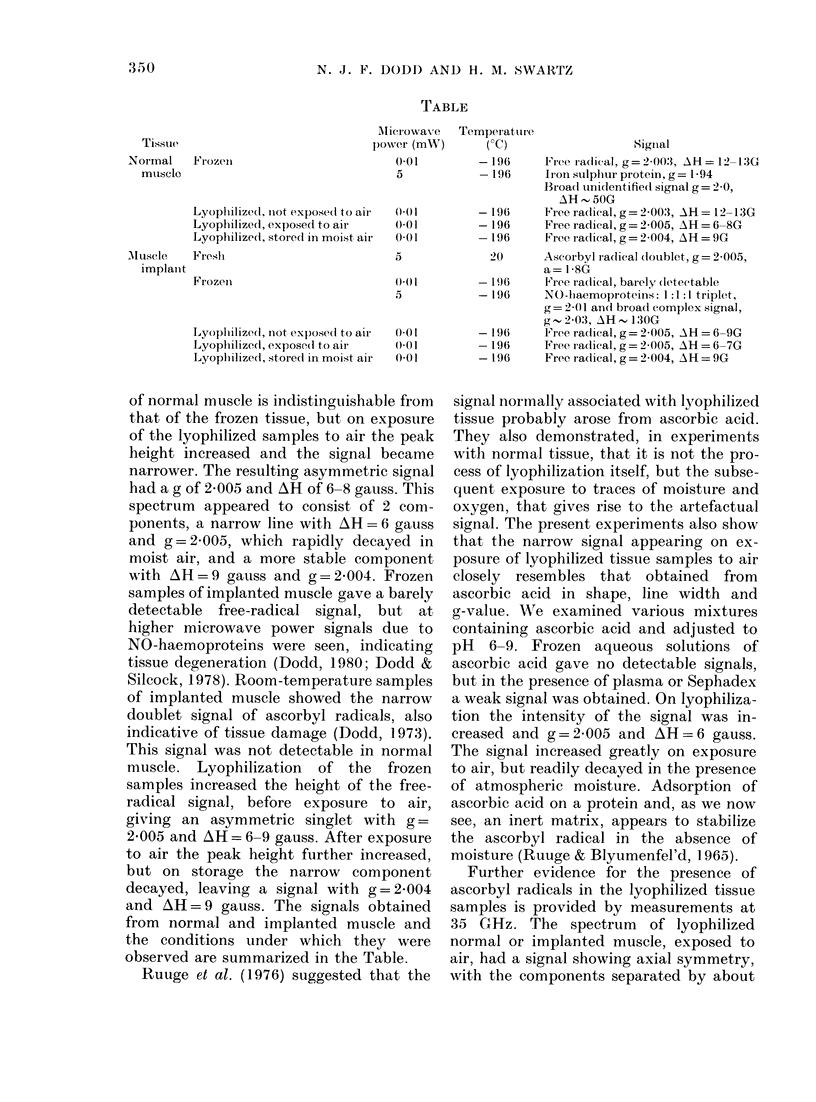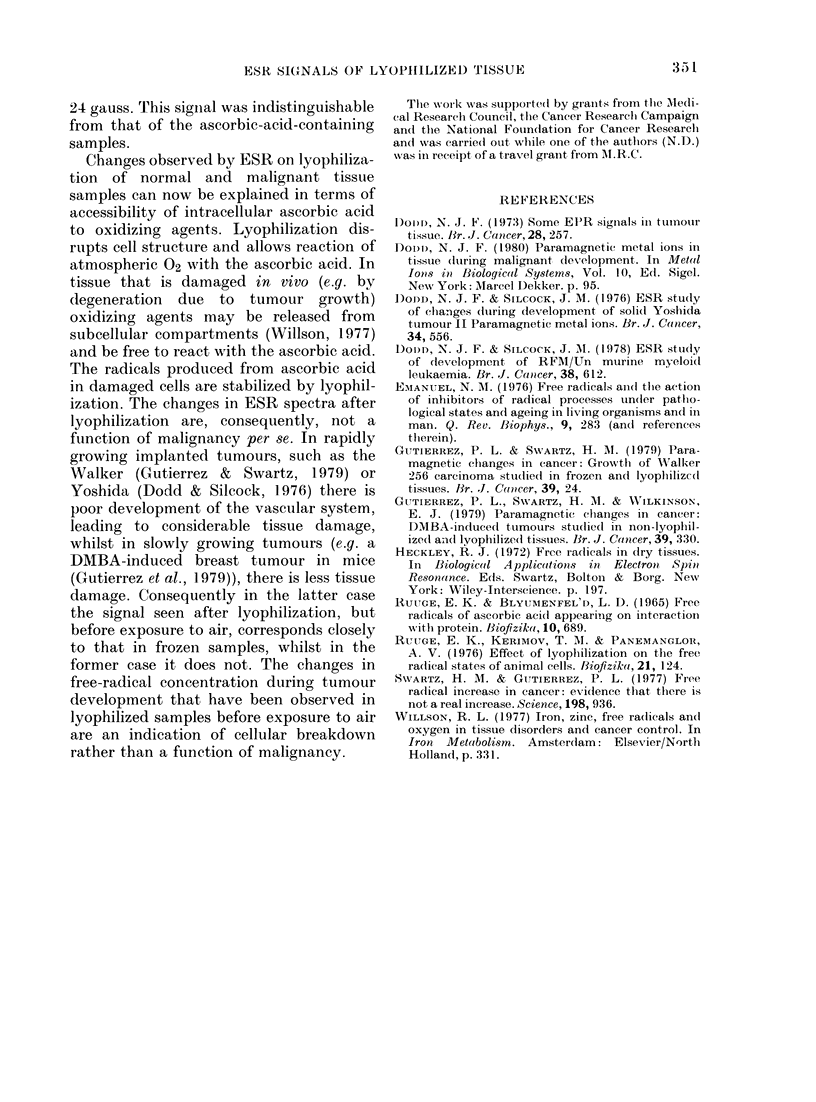# ESR signals of lyophilized tissue.

**DOI:** 10.1038/bjc.1980.238

**Published:** 1980-08

**Authors:** N. J. Dodd, H. M. Swartz


Br. J. Cancer (1980) 42, 349

***Short Communication***

### ESR SIGNALS OF LYOPHILIZED TISSUE

N. J. F. DODD AND H. M. SWARTZ*†

*From the Paterson Laboratories, Christie Hospital and Holt Radium Institute, Manchester, and the *National Biomedical ESR Center, Medical College of Wisconsin, Milwaukee, Wisconsin 53226, U.S.A.*

Received 8 February 1980 Accepted 30 April 1980

DURING MALIGNANT DEVELOPMENT, systematic changes in free-radical content have been observed in lyophilized tissues from experimental animals carrying several different tumours and leukaemias (Emanuel, 1976). However, the free-radical content of lyophilized preparations does not necessarily represent that of the samples before drying (Heckley, 1972). Recently, careful comparisons have been made between the free-radical ESR signals in both normal and malignant tissues, before and after lyophilization (Swartz & Gutierrez, 1977; Gutierrez & Swartz, 1979; Gutierrez *et al.*, 1979). Under strictly controlled conditions, in which samples were lyophilized whilst avoiding exposure to air, reproducible data could be obtained, but the signals in lyophilized tumour samples bore no simple relationship to those seen before lyophilization. Here we report further studies of lyophilized and frozen tissues, using normal and implanted rat muscle as model systems, and show that the discrepancies between frozen and lyophilized samples can be explained in terms of cellular damage and the production of ascorbyl radicals.

Normal muscle tissue was taken from the hind legs of adult male Sprague–Dawley rats, immediately after death from cervical dislocation, and was implanted s.c. into the flanks of anaesthetized Sprague–Dawley rats. After periods of time from 1 to 10 days after implantation, the rats were killed and the implants, together with samples of normal muscle, were removed. Tissue was packed into 4mm internal-diameter (i.d.) precision-bore moulds (for 9·3GHz ESR measurement) or calibrated 1mm i.d. quartz tubes (for 35 GHz), quickly frozen and stored in liquid $N_2$ until ESR analysis. These samples were examined in the frozen state and then lyophilized, while retaining the same configuration (Swartz & Gutierrez, 1977). Samples were examined at 9·3 GHz in liquid $N_2$ in a fingertip Dewar, using a Varian E (Century) spectrometer with $TE_{102}$ cavity. A 100kHz modulation of amplitude 4 gauss and incident microwave power of 0·01 mW were used to study free radicals and 2·5 gauss and 5 mW respectively to study metal ions. 35GHz measurements were made at a temperature of $-140°C$, using a Varian E-9 spectrometer equipped with a variable-temperature accessory (E-268) and $TE_{011}$ cavity. The modulation amplitude was 4 gauss and the incident microwave power 0·006 mW.

Frozen samples of normal muscle, examined at X-band at a low microwave power, showed a weak free-radical signal with a line width ($\Delta H$) of 12–13 gauss and g-value of about 2·003. At higher power the free-radical signal saturated, revealing a broader underlying signal in addition to an iron-sulphur protein signal at $g = 1{\cdot}94$. After lyophilization, the free-radical signal

† Present address: University of Illinois-Urbana, Medical Science Building, Urbana, Illinois 61801, U.S.A.

TABLE

| Tissue | | Microwave power (mW) | Temperature (°C) | Signal |
|---|---|---|---|---|
| Normal muscle | Frozen | 0·01 | − 196 | Free radical, g = 2·003, ΔH = 12–13G |
| | | 5 | − 196 | Iron sulphur protein, g = 1·94 Broad unidentified signal g = 2·0, ΔH ~ 50G |
| | Lyophilized, not exposed to air | 0·01 | − 196 | Free radical, g = 2·003, ΔH = 12–13G |
| | Lyophilized, exposed to air | 0·01 | − 196 | Free radical, g = 2·005, ΔH = 6–8G |
| | Lyophilized, stored in moist air | 0·01 | − 196 | Free radical, g = 2·004, ΔH = 9G |
| Muscle implant | Fresh | 5 | 20 | Ascorbyl radical doublet, g = 2·005, a = 1·8G |
| | Frozen | 0·01 | − 196 | Free radical, barely detectable |
| | | 5 | − 196 | NO-haemoproteins: 1:1:1 triplet, g = 2·01 and broad complex signal, g ~ 2·03, ΔH ~ 130G |
| | Lyophilized, not exposed to air | 0·01 | − 196 | Free radical, g = 2·005, ΔH = 6–9G |
| | Lyophilized, exposed to air | 0·01 | − 196 | Free radical, g = 2·005, ΔH = 6–7G |
| | Lyophilized, stored in moist air | 0·01 | − 196 | Free radical, g = 2·004, ΔH = 9G |

of normal muscle is indistinguishable from that of the frozen tissue, but on exposure of the lyophilized samples to air the peak height increased and the signal became narrower. The resulting asymmetric signal had a g of 2·005 and ΔH of 6–8 gauss. This spectrum appeared to consist of 2 components, a narrow line with ΔH = 6 gauss and g = 2·005, which rapidly decayed in moist air, and a more stable component with ΔH = 9 gauss and g = 2·004. Frozen samples of implanted muscle gave a barely detectable free-radical signal, but at higher microwave power signals due to NO-haemoproteins were seen, indicating tissue degeneration (Dodd, 1980; Dodd & Silcock, 1978). Room-temperature samples of implanted muscle showed the narrow doublet signal of ascorbyl radicals, also indicative of tissue damage (Dodd, 1973). This signal was not detectable in normal muscle. Lyophilization of the frozen samples increased the height of the free-radical signal, before exposure to air, giving an asymmetric singlet with g = 2·005 and ΔH = 6–9 gauss. After exposure to air the peak height further increased, but on storage the narrow component decayed, leaving a signal with g = 2·004 and ΔH = 9 gauss. The signals obtained from normal and implanted muscle and the conditions under which they were observed are summarized in the Table.

Ruuge *et al.* (1976) suggested that the signal normally associated with lyophilized tissue probably arose from ascorbic acid. They also demonstrated, in experiments with normal tissue, that it is not the process of lyophilization itself, but the subsequent exposure to traces of moisture and oxygen, that gives rise to the artefactual signal. The present experiments also show that the narrow signal appearing on exposure of lyophilized tissue samples to air closely resembles that obtained from ascorbic acid in shape, line width and g-value. We examined various mixtures containing ascorbic acid and adjusted to pH 6–9. Frozen aqueous solutions of ascorbic acid gave no detectable signals, but in the presence of plasma or Sephadex a weak signal was obtained. On lyophilization the intensity of the signal was increased and g = 2·005 and ΔH = 6 gauss. The signal increased greatly on exposure to air, but readily decayed in the presence of atmospheric moisture. Adsorption of ascorbic acid on a protein and, as we now see, an inert matrix, appears to stabilize the ascorbyl radical in the absence of moisture (Ruuge & Blyumenfel'd, 1965).

Further evidence for the presence of ascorbyl radicals in the lyophilized tissue samples is provided by measurements at 35 GHz. The spectrum of lyophilized normal or implanted muscle, exposed to air, had a signal showing axial symmetry, with the components separated by about

24 gauss. This signal was indistinguishable from that of the ascorbic-acid-containing samples.

Changes observed by ESR on lyophilization of normal and malignant tissue samples can now be explained in terms of accessibility of intracellular ascorbic acid to oxidizing agents. Lyophilization disrupts cell structure and allows reaction of atmospheric $O_2$ with the ascorbic acid. In tissue that is damaged *in vivo* (*e.g.* by degeneration due to tumour growth) oxidizing agents may be released from subcellular compartments (Willson, 1977) and be free to react with the ascorbic acid. The radicals produced from ascorbic acid in damaged cells are stabilized by lyophilization. The changes in ESR spectra after lyophilization are, consequently, not a function of malignancy *per se*. In rapidly growing implanted tumours, such as the Walker (Gutierrez & Swartz, 1979) or Yoshida (Dodd & Silcock, 1976) there is poor development of the vascular system, leading to considerable tissue damage, whilst in slowly growing tumours (*e.g.* a DMBA-induced breast tumour in mice (Gutierrez *et al.*, 1979)), there is less tissue damage. Consequently in the latter case the signal seen after lyophilization, but before exposure to air, corresponds closely to that in frozen samples, whilst in the former case it does not. The changes in free-radical concentration during tumour development that have been observed in lyophilized samples before exposure to air are an indication of cellular breakdown rather than a function of malignancy.

The work was supported by grants from the Medical Research Council, the Cancer Research Campaign and the National Foundation for Cancer Research and was carried out while one of the authors (N.D.) was in receipt of a travel grant from M.R.C.

### REFERENCES

DODD, N. J. F. (1973) Some EPR signals in tumour tissue. *Br. J. Cancer*, **28**, 257.

DODD, N. J. F. (1980) Paramagnetic metal ions in tissue during malignant development. In *Metal Ions in Biological Systems*, Vol. 10, Ed. Sigel. New York: Marcel Dekker. p. 95.

DODD, N. J. F. & SILCOCK, J. M. (1976) ESR study of changes during development of solid Yoshida tumour II Paramagnetic metal ions. *Br. J. Cancer*, **34**, 556.

DODD, N. J. F. & SILCOCK, J. M. (1978) ESR study of development of RFM/Un murine myeloid leukaemia. *Br. J. Cancer*, **38**, 612.

EMANUEL, N. M. (1976) Free radicals and the action of inhibitors of radical processes under pathological states and ageing in living organisms and in man. *Q. Rev. Biophys.*, **9**, 283 (and references therein).

GUTIERREZ, P. L. & SWARTZ, H. M. (1979) Paramagnetic changes in cancer: Growth of Walker 256 carcinoma studied in frozen and lyophilized tissues. *Br. J. Cancer*, **39**, 24.

GUTIERREZ, P. L., SWARTZ, H. M. & WILKINSON, E. J. (1979) Paramagnetic changes in cancer: DMBA-induced tumours studied in non-lyophilized and lyophilized tissues. *Br. J. Cancer*, **39**, 330.

HECKLEY, R. J. (1972) Free radicals in dry tissues. In *Biological Applications in Electron Spin Resonance*. Eds. Swartz, Bolton & Borg. New York: Wiley-Interscience. p. 197.

RUUGE, E. K. & BLYUMENFEL'D, L. D. (1965) Free radicals of ascorbic acid appearing on interaction with protein. *Biofizika*, **10**, 689.

RUUGE, E. K., KERIMOV, T. M. & PANEMANGLOR, A. V. (1976) Effect of lyophilization on the free radical states of animal cells. *Biofizika*, **21**, 124.

SWARTZ, H. M. & GUTIERREZ, P. L. (1977) Free radical increase in cancer: evidence that there is not a real increase. *Science*, **198**, 936.

WILLSON, R. L. (1977) Iron, zinc, free radicals and oxygen in tissue disorders and cancer control. In *Iron Metabolism*. Amsterdam: Elsevier/North Holland, p. 331.